# The effect of allometric partitioning on herbivory tolerance in four species in South China

**DOI:** 10.1002/ece3.5651

**Published:** 2019-10-02

**Authors:** Zhe‐Xuan Fan, Bao‐Ming Chen, Hui‐Xuan Liao, Guo‐Hao Zhou, Shao‐Lin Peng

**Affiliations:** ^1^ State Key Laboratory of Biocontrol and Guangdong Key Laboratory of Plant Resources School of Life Sciences Sun Yat‐Sen University Guangzhou China; ^2^ School of Life Sciences Guizhou Normal University Guiyang Guizhou Province China

**Keywords:** allometric exponent, biomass allocation, herbivory tolerance

## Abstract

Herbivory tolerance can offset the negative effects of herbivory on plants and plays an important role in both immigration and population establishment. Biomass reallocation is an important potential mechanism of herbivory tolerance. To understand how biomass allocation affects plant herbivory tolerance, it is necessary to distinguish the biomass allocations resulting from environmental gradients or plant growth. There is generally a tight balance between the amounts of biomass invested in different organs, which must be analyzed by means of an allometric model. The allometric exponent is not affected by individual growth and can reflect the changes in biomass allocation patterns of different parts. Therefore, the allometric exponent was chosen to study the relationship between biomass allocation pattern and herbivory tolerance. We selected four species (*Wedelia chinensis*, *Wedelia trilobata*, *Merremia hederacea*, and *Mikania micrantha*), two of which are invasive species and two of which are accompanying native species, and established three herbivory levels (0%, 25% and 50%) to compare differences in allometry. The biomass allocation in stems was negatively correlated with herbivory tolerance, while that in leaves was positively correlated with herbivory tolerance. Furthermore, the stability of the allometric exponent was related to tolerance, indicating that plants with the ability to maintain their biomass allocation patterns are more tolerant than those without this ability, and the tendency to allocate biomass to leaves rather than to stems or roots helps increase this tolerance. The allometric exponent was used to remove the effects of individual development on allocation pattern, allowing the relationship between biomass allocation and herbivory tolerance to be more accurately explored. This research used an allometric model to fit the nonlinear process of biomass partitioning during the growth and development of plants and provides a new understanding of the relationship between biomass allocation and herbivory tolerance.

## Graphical abstract

Contrast to the biomass ratio, allometric index is not affected by ontogenetic drift. Partition and variation of partition two aspects affect herbivore tolerance. Plants which maintain their biomass allocation patterns are more tolerant than others. Plants allocate to leaves rather than stems or roots helps to increase tolerance.

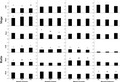

## 1 INTRODUCTION

Herbivory typically has a negative effect on plant fitness, and plants are pressured to increase levels of defence (Strauss & Agrawal, 1999). Tolerance is an important plant defence strategy in which plants compensate for tissue loss to counteract the negative effects of herbivory. The defence strategies of plants may change their ability to withstand herbivores (Anderson & Briske, 1995; Stowe, Marquis, Hochwender, & Simms, 2000). Tolerance also plays an important role in community diversity and population establishment (Mariotte, Buttler, Kohler, Gilgen, & Spiegelberger, 2013), and increased herbivory tolerance is thought to be one of the reasons that some species have higher capacities to become invasive (Fornoni, 2011; Wang et al., 2011; Zou, Siemann, Rogers, & DeWalt, 2008). Tolerance is related to biomass allocation pattern, but plants have a remarkable capacity to coordinate the growth of their organs, such that there is generally a tight balance between the amounts of biomass invested in different organs, which requires analysis by means of an allometric model. Therefore, additional research is needed to determine the mechanisms of allometric partitioning that enable plants to tolerate herbivory.

Much research has explored the mechanisms of plant tolerance (Rosenthal & Kotanen, 1994; Strauss & Agrawal, 1999; Tiffin, 2000). The mechanisms underlying tolerance are potentially complex and can involve numerous plant traits that facilitate recovery, such as an increase in the photosynthetic rate after herbivory (Stowe et al., 2000; Trumble, Kolodny‐Hirsch, & Ting, 1993), apical meristem activity after damage (Suwa & Maherali, 2008; Wise & Abrahamson, 2007), and plant phenological changes, such as delays in growth, flowering, and fruit production (Tiffin, 2000). The potential tolerance of plants is also affected by changes in their composition as well as stored resources, resource reallocation, and architecture (Moreira, Zas, & Sampedro, 2012; Stevens, Kruger, & Lindroth, 2008); all of these traits contribute to the tolerance of herbivores.

The key mechanism of herbivory tolerance in plants is the redistribution of resources, and biomass allocation is the central driver of plant life‐history strategies (Müller, Schmid, & Weiner, 2000; Weiner, 2004) and the basis of the environmentally sensitive response strategy employed by plants. Research on the relationship between biomass allocation and herbivory tolerance has mainly focused on two aspects: (a) how biomass allocation patterns influence herbivory tolerance and (b) how the capacity to alter biomass allocation patterns in response to herbivores influences herbivory tolerance. Some studies based on variation in biomass partitioning have shown that species with the ability to maintain similar root‐to‐shoot ratios after herbivory are more tolerant than those without this ability (Ashton & Lerdau, 2008; Lieurance & Cipollini, 2013). Additionally, plants with higher root‐to‐shoot ratios are more tolerant than those with lower root‐to‐shoot ratios (Barton, 2013; Hochwender, Marquis, & Stowe, 2000; Mabry & Wayne, 1997; Rivera et al., 2012), likely due to stored resources in roots and greater nutrient uptake, both of which are important to support the increase in growth following defoliation (Moreira et al., 2012).

Biomass allocation is an important mechanism of herbivory tolerance (Gassmann, 2004), but there are disagreements related to the methods used to measure variation in biomass allocation. Many related studies have used the biomass ratios of different plant parts to represent biomass allocation. However, it is difficult to distinguish the source of the variation: environmental impacts or ontogenetic drift (Huang et al., 2009; McConnaughay & Coleman, 1999; Moriuchi & Winn, 2005). Numerous studies have indicated that the biomass allocation patterns of plant organs are size‐dependent (McConnaughay & Coleman, 1999; Niinemets, 2004; Wright & McConnaughay, 2002). However, many other studies have used proportional changes to reflect herbivory tolerance or compare the tolerances of different species (Araminiene, Varnagiryte‐Kabašinskiene, & Stakenas, 2017; Lurie, Barton, & Daehler, 2017; Stevens et al., 2008; Wang, Bezemer, van der Putten, Brinkman, & Biere, 2018; Wang et al., 2017; Zvereva, Lanta, & Kozlov, 2010). The ratios used to test biological hypotheses may change with plant size and cannot accurately measure the relationship between herbivory tolerance and biomass allocation. For example, the results of a previous study indicated that the tolerance and biomass allocation of seedlings were different from those of mature plants (Barton, 2013), probably because the ratio masked the difference in biomass allocation patterns among plants of different sizes.

The relationships among the parts of an organism are often nonlinear, and most organisms grow allometrically rather than isometrically over time (Jasienski & Bazzaz, 1999; Niklas & Enquist, 2002a, 2002b; Sack, Marañón, & Grubb, 2002; Weiner, 2004; Weiner et al., 2009). Weiner (2004) argued that the relationship between growth and allocation should be quantified by allometry and not by ratios or proportions. Metabolic theory provides a framework that focuses on the relationship between body size and growth‐related phenomena, including metabolic allocation and biomass partitioning (Enquist, Brown, & West, 1998; Enquist & Niklas, 2002; Enquist, West, Charnov, & Brown, 1999; Niklas & Enquist, 2002a, 2002b; West, Brown, & Enquist, 1997, 1999). According to the theory, the metabolic rate scales with body size based on a 3/4 scaling exponent in animals and plants, leading to the predictions that leaf biomass will scale as the 3/4 power of stem biomass and root biomass and that stem biomass and root biomass will scale isometrically with respect to each other. However, allometric exponents are not constant, instead varying with different factors (Chen & Li, 2003; Chu et al., 2010; Enquist et al., 2007; Mori et al., 2010; Reich, Tjoelker, Machado, & Oleksyn, 2006; Zhang, Wang, Ji, Fan, & Deng, 2011). Therefore, we used an allometric model to distinguish the roles of body size and different patterns in the allocation response to the environment, which furthers our understanding of the herbivory tolerance of plants.

An allometric model was used to study the relationship between biomass allocation patterns and herbivory tolerance. We selected four species from South China, including two common invasive species and two local species with similar growth forms. We aimed to determine whether plant biomass allocation patterns have effects on herbivory tolerance. Thus, the study focused on two aspects: whether partitioning pattern influences tolerance and whether variation in biomass partitioning influences tolerance.

## 2 MATERIALS AND METHODS

### 2.1 Experimental design

A common garden was established for potted plants on the campus of Sun Yat‐sen University, Guangzhou, China. The experiment included 12 combinations of three levels of defoliation (0%, 25% and 50%) and four species (*Wedelia trilobata*, *Mikania micrantha*, *Wedelia chinensis*, and *Merremia hederacea*) and was conducted with a split plot design to minimize asymmetric competition for light. The 12 combinations were replicated across 25 blocks for a total of 300 plants. Each pot contained only one plant, and pots were placed adjacent to each other with 0.5 m between pairs. Rhizomes were used in our experiments, and the 12 combinations were replicated across more than 25 rhizomes to ensure that we had sufficient plants. All of the plants were planted on September 3, 2014, and harvested on December 25, 2014; we cut off shoots and then separated them into leaves and stems, and the roots were collected from the soil and rinsed. Plants were dried to a constant weight at 60°C.

Invasive plants may be more tolerant than native species to herbivores (Ashton & Lerdau, 2008; Wang et al., 2011; Zou, Siemann, et al., 2008). Thus, four species of plants (*W. chinensis*, *W. trilobata*, *M. hederacea*, and *M. micrantha*) native or invasive to South China were selected. *Wedelia trilobata* and *M. micrantha* are invasive species that are widely distributed in disturbed areas. *Mikania micrantha* grows rapidly and reproduces by seed production and vegetative propagation. *Wedelia chinensis* and *M. hederacea* are native species that are mainly distributed throughout South China. *Merremia hederacea* and *M. micrantha* are perennial herbaceous twisting vines, and *M. hederacea* frequently appears with *M. micrantha*. *Wedelia chinensis* and *W. trilobata* are perennial herbs with creeping rootstocks, and they have low seed production and spread by vegetative propagation.

### 2.2 Defoliation and tolerance measurements

We used jasmonic acid combined with clipping to simulate herbivory. Two‐thirds of the plants were clipped, and one‐third was left as an undefoliated control. We removed 25% of 1/3 of the plants and 50% of the other 1/3, and all measurements of these samples were taken in October and November of 2014. Herbivory was simulated by using scissors, and 1 mmol/L jasmonic acid (M111207; Aladdin Chemical Co.) was sprayed onto the plants that were clipped (Baldwin, 1996). The plants that had not been clipped were sprayed until dripping with solvent (methanol and distilled water) instead of jasmonic acid. Jasmonic acid is a natural elicitor of herbivory defences and induces an herbivory response (Thaler, Stout, Karban, & Duffey, 1996).

Tolerance was assessed by comparing the mean relative growth of the defoliated plants of a given species to that of the undefoliated plants of the same species (Stevens et al., 2008; Stowe et al., 2000; Strauss & Agrawal, 1999). Tolerance was defined as the growth difference between damaged and undamaged plants (Hochwender et al., 2000; Tiffin, Rausher, Associate Editors: Thomas, & Joy, 1999).

### 2.3 Statistical analyses

We compared the herbivory tolerances of the different species and analyzed the allocation of plant biomass and the linear relationship between the allocation of plant biomass and herbivory tolerance of the plants. Tolerance was calculated as the biomass difference between damaged and undamaged plants at the end of the experiment (Strauss & Agrawal, 1999). We compared the biomass of damaged plants to the average biomass of undamaged plants of the same species. The difference in tolerance was analyzed using a linear mixed model in which species and the degree of defoliation were considered fixed effects and block was considered a random effect. We fit linear mixed‐effects models using the “Eigen” and S4 (lme4) packages in R (R version 3.3.0; R Foundation for Statistical Computing).

A standardized major axis (SMA) regression analysis was used to test the log_10_ *Y*‐log_10_ *X* scaling relationship. The allometric exponent (*b*) was computed using the formula *Y* = *aX^b^*, where *a* is a normalization constant that varies with *Y* and the kind of organism, which was changed to log_10_ *Y* = *b*•log_10_ *X* + log_10_ *a*. The SMA slope heterogeneity for biomass allocation was determined using the Standardized Major Axis Estimation and Testing Routines (SMATR) package of R (Bates, Machler, Bolker, & Walker, 2015; Warton, Duursma, Falster, & Taskinen, 2012). SMA regression was used to explore the relationships between different plant organs and whole plants, where different slopes represented the relationship between biomass allocation and herbivory. In this study, we wanted to test for variation in biomass allocation among species and within species exposed to different treatments. Different slopes indicate that the relationship between the given variable and biomass allocation is influenced by herbivory. Equal slopes among treatments indicate that the relationship between the given variable and biomass allocation remains the same at different herbivory levels, that is, biomass allocation is a function of only plant size.

To assess the relationship between tolerance and biomass allocation, we analyzed the relationships between tolerance score and the slope of the allometric relationship of plant organs and whole plants and calculated the correlation coefficients between them. To compare the biomass ratio with the allometric index, we analyzed variation in the ratios of plant‐part biomass to whole‐plant biomass and used linear regression to determine the relationships between tolerance score and the ratios.

## 3 RESULTS

### 3.1 Tolerance

**Figure 1:**
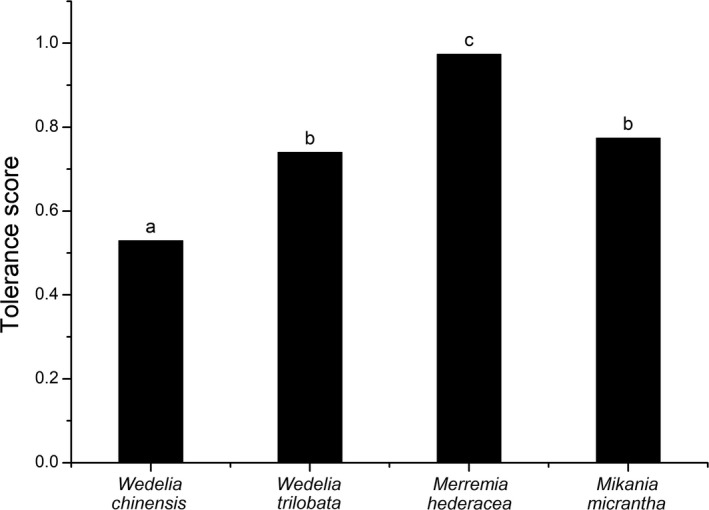
Comparison of the tolerance scores for four species; each bar represents the average tolerance score of two levels for one species. A two‐factor linear mixed model was used to assess the species and herbivore levels (95% confidence interval)

The two invasive plants were more tolerant than the native plants, and the vines were more tolerant than the plants with creeping rootstocks (Figure 1). Among the four species, *M. hederacea* had the highest tolerance score, and *W. chinensis* showed the lowest tolerance score. There was no significant difference between the two invasive plants, namely, *M. micrantha* and *W. trilobata*. In terms of life form, the tolerance scores of the vines were approximately 38% higher than those of the plants with creeping rootstocks. Our results supported the idea that invasive species are not always more tolerant than native species to herbivory (Ashton & Lerdau, 2008; Wang et al., 2011; Zou, Siemann, et al., 2008). The tolerance of *W. trilobata* was higher than that of *W. chinensis* (by approximately 40%), but for the vines, the tolerance of the native species *M. micrantha* was higher than that of the invasive species (by 26%).

### 3.2 Allometric exponent

We compared the allometric exponents of different organs to those of the whole plant for the four species, and the exponents ranged widely from 0.79 to 1.4 (Table 1). The largest allometric exponent for leaves occurred in *M. hederacea*, while the smallest occurred in *W. chinensis*. Conversely, the largest allometric exponent for stems occurred in *W. chinensis*, while the smallest occurred in *W. chinensis*. The largest allometric exponent for roots occurred in *M. micrantha*, while the smallest occurred in *W. trilobata*. The allometric relationships between leaves and whole plants differed markedly between all species pairs except *M. micrantha* and *M. hederacea*. Similarly, the stem exhibited similar trends in all four species except *M. micrantha* and *W. trilobata*. Conversely, the allometric relationship between roots and whole plants differed only between *M. micrantha* and *W. chinensis*.

**Table 1:** Allometric relationship between log (leaf, root, stem) and log (whole‐plant biomass) for individuals of four species, *b* is the of slope two various (allometry exponent)

	Leaf			Stem			Root		
	*b*	95% CI	Slope_test_*p*	*b*	95% CI	Slope_test_*p*	*b*	95% CI	Slope_test_*p*
*Wedelia chinensis*	0.851752	0.798–0.9092	<.001	1.408047	1.3534–1.465	<.001	0.786724	0.7186–0.8614	<.001
*Wedelia trilobata*	0.958229	0.9045–1.0151	<.001	1.17728	1.132–1.2244	<.001	0.856711	0.7556–0.9714	.0164
*Merremia hederacea*	1.264302	1.0363–1.5424	<.001	0.998536	0.9324–1.0693	.9661	0.873701	0.7729–0.9877	.0313
*Mikania micrantha*	1.20412	1.1334–1.2793	<.001	1.104046	1.0391–1.1730	.0017	0.911154	0.8183–1.0145	.0889

Note

Individuals in the undamaged groups were not included in the analysis.

**Figure 2:**
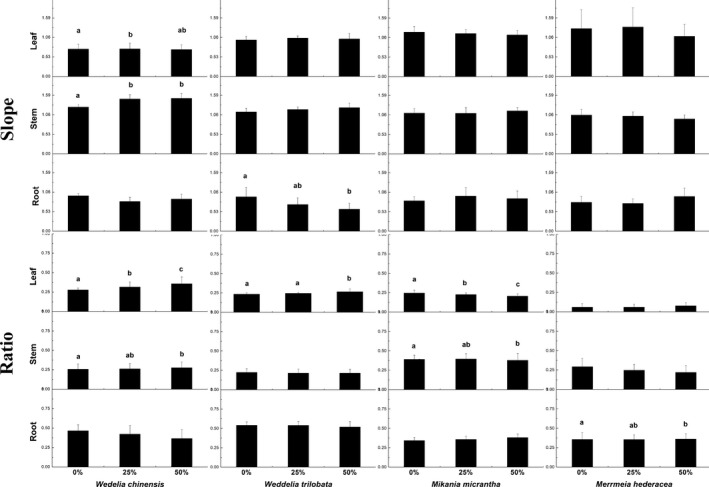
Values of allometric exponent and slope in different defoliation level. Bars represent the allometric exponent (slope)/ratio for the parts and whole plants to leaves, roots, and stems between damaged and undamaged groups for four species

In terms of life form, there were no significant differences between the two vine plants, but the allometry of biomass allocation to leaves, stems, and roots by the plants with creeping rootstocks was affected by the treatment (Figure 2). The allometry of biomass allocation to leaves and stems differed between the treatment and the control for *W. chinensis*. For the invasive plants, the allocation to leaves and stems in *W. trilobata* was not affected by the treatment, but the allocation to roots differed between the treatment and the control, with less biomass allocated to roots as herbivory increased. In the two vine plants, there were no significant differences between the treatment and the control.

### 3.3 Relationship between tolerance and the allometric exponent

**Figure 3:**
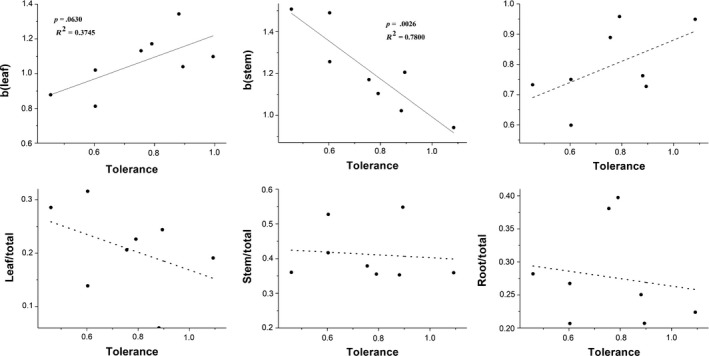
Correlations between tolerance (difference between the biomass of damaged and undamaged plants) and the allometric relationship ratios of plant parts and whole plants to leaves, roots, and stems for four species. *b* is the of slope two various (allometry exponent), the each point represents the mean response of the tolerance and allometric slope for each defoliation level of a single species. Solid line represents there are significant differences for test of the regression, dashed line represents there is no significant difference for test of the regression

The relationship between the allometry of biomass allocation and tolerance was regressed for the four species and two treatments. The allometric exponent of biomass allocation to leaves was positively correlated with the tolerance score, but that to stems was negatively correlated with tolerance. There was no significant relationship between root biomass allocation and tolerance. The ratio and tolerance results were similar, but none of the relationships were significant (Figure 3).

## 4 DISCUSSION

According to our results, the allometric scaling relationship is related to the herbivory tolerance score, and there is no correlation between the partitioning ratio and herbivory tolerance (Figure 3). Different parts have distinct effects on tolerance. Herbivory tolerance is positively correlated with leaf mass allocation and negatively correlated with stem mass allocation but is not related to root biomass. The partitioning of biomass in roots is related to the herbivory tolerance of herbs (Hochwender et al., 2000; Moreira et al., 2012); however, tolerance is also related to stems and leaves (Pratt, Rayamajhi, Van, Center, & Tipping, 2005; Stevens et al., 2008). Stevens et al. (2008) showed that herbivory tolerance was positively correlated with stem biomass allocation and negatively correlated with root biomass allocation in woody plants. Our results are different from the results of research on herbs and woody plants, and the possible causes of this difference are that the previous studies ignored the effects of body size on the biomass partitioning ratio and mainly focused on woody plants and herbs, whereas little such research has been conducted on vines. The mechanisms used to tolerate herbivore damage include photosynthetic activity (Gassmann, 2004; Li, Luo, Tian, Peng, & Zhou, 2012; Li, Tian, Luo, Dai, & Peng, 2012) and stored reserves (Boege, 2005; Newingham, Callaway, & BassiriRad, 2007; Thomas, Abbott, & Moloney, 2017; Wang et al., 2018, 2017). In response to herbivore damage, plants allocate more resources to photosynthesis, which leads to greater biomass allocation to leaves, indicating that vines tend to improve their photosynthetic activities to enhance herbivory tolerance. Stems are thought to be the primary source of nonstructural carbohydrates (TNCs) in plants (Barton, 2016; Myers & Kitajima, 2007; Willaume & Pagès, 2011). Less partitioning to stems indicates the utilization of TNCs (Chapin & McNaughton, 1989; Van Der Heyden & Stock, 1996) and reduces the limitation caused by transfer of resources from undamaged areas to damaged areas in longer stems. Divergence in the relationship between biomass partitioning and herbivory tolerance is also due to differences in environmental factors, life forms, conditions, and indicators.

Herbivores influence plant biomass partitioning to various degrees. Niklas and Enquist (2002a, 2002b) used allometric theory to predict that the scaling relationship of three organic growth rates was isometric, and an allometric scaling relationship was used to describe the biomass partitioning for these three parts. Many environmental factors, including biological factors and nonbiological factors, can influence the allometric exponent (Chu et al., 2010; Deng et al., 2008; Lin, Berger, Grimm, Huth, & Weiner, 2013), and the allometric scaling relationship between body size and metabolic rate is not fixed (Chu et al., 2010; Glazier, 2010). Variation in the allometric relationships between the three parts and plant biomass can reflect variation in patterns of biomass allocation under changing conditions. Thus, our results (Figure 2) indicated that plants with less allometric exponent variation after damage exhibited less variation in their biomass allocation pattern; these plants also had higher tolerance than those with more allometric exponent variation. A change in the allometry of biomass allocation to leaves and stems occurred in *W. chinensis*, but the biomass allocation to roots in *W. trilobata* differed between treatments. The allometric exponent of the vines was not influenced by herbivory, potentially because species with higher tolerance scores often have stronger abilities to transfer resources from undamaged areas to damaged areas (Irwin, Galen, Rabenold, Kaczorowski, & McCutcheon, 2008) and recover their original allocation pattern (Ashton & Lerdau, 2008; Lieurance & Cipollini, 2013). Our results confirm the hypothesis in which species (except *W. chinensis*) reallocate biomass to different parts to maintain a similar structure after damage, and *W. chinensis* has a lower tolerance than the other species. The results of the regression revealed no relationship between root partitioning and tolerance. The root biomass of *W. trilobata* was also affected by herbivory, but this species has a relatively high tolerance score, which also illustrates that herbivore tolerance is not influenced by variation in the partitioning pattern of roots.

Our results suggested that the allometric exponent reflects the relationship between biomass allocation pattern and herbivory tolerance better than does the biomass ratio. Allocation is size‐dependent, and allocation patterns can be thought of in an allometric way and are a function of body size (Price et al., 2012; Sibly, Brown, & Kodric‐Brown, 2012). Ratios were used to reflect the allocation patterns of plants in previous research, but the changes in the ratios of different parts observed here are attributed to changes in body size or allocation patterns, and we cannot be certain that the imposed treatment did not influence the allocation patterns of the plants. Weiner et al. (2009) explored the relationship between vegetative and reproductive structures using an allometric model and found that the reproductive biomass ratio changed at different nutrient levels; however, there was no variation in the allometric exponent. This result indicates that the change in plant size caused the change in the reproductive biomass ratio at different nutrient levels, and the invariance of the allometric exponent reflects the invariance of the allocation pattern. A change in the biomass ratio cannot reflect the influence of nutrient levels on allocation patterns; similarly, when plants experience herbivory, the ratio changes may mask the changes in plant biomass allocation to different parts and the relationships between different parts. The patterns of variation in the allometric exponent and tolerance were similar, but the ratio results did not change in line with the tolerance patterns (Figure 1, Table 1). Our results suggest that an allometric model is better than ratios to reflect the herbivory tolerance of plants.

Ontogenetic drift and response to the environment cooperate to influence the development of organs (Niklas, 2006), and environmental selection can change the developmental trajectories of organs and delay their growth in resource‐poor environments. Therefore, it is necessary to distinguish the changes in biological characteristics caused by changes in ontogenetic trajectory from those caused by changes in plant size. The allometric relationship of biomass partitioning can reflect the effects of environmental factors on plants, and some studies have shown that the allometric trajectory is plastic (Weiner, 2004). Numerous studies have evaluated whether allocation patterns are influenced by experimental measurements of allometry (Achten et al., 2010; Guo et al., 2012; Hulshof, Stegen, Swenson, Enquist, & Enquist, 2012; Poorter, 2001; Preston & Ackerly, 2003; Qin, Weiner, Qi, Xiong, & Li, 2013). Xie, Tang, Wang, Xu, and Li (2012) discussed the influence of soil texture on plant biomass allocation. Guo et al. (2012) compared the allometric relationships of reproductive and vegetative mass for 24 species of *Pedicularis* at different elevations, reporting fundamental changes in the costs and benefits of increased vegetative biomass with elevation.

Invasive species were not always more tolerant than native species in our experiment. The enemy release hypothesis (ERH) and evolution of increased competitive ability (EICA) hypothesis (Blossey & Notzold, 1995; Keane & Crawley, 2002; Shea & Chesson, 2002; Williamson, 1996) suggest that invasive plants, which escape from their enemies, are often more tolerant than native species (Ashton & Lerdau, 2008; Wang et al., 2018, 2011; Zou, Rogers, & Siemann, 2008; Zou, Siemann, et al., 2008). However, some studies have drawn different conclusions. Lurie et al. (2017) researched the resistance and tolerance of 12 groups of native, invasive, and naturalized vines and found that invasive vines were more tolerant than native and naturalized relatives of simulated herbivory. Our results also showed that invasive plants were more tolerant than native plants on average, but the invasive species did not always have higher tolerance scores than the native species. *Merremia hederacea* was more tolerant than *M. micrantha*, but the native species *W. trilobata* was much more tolerant than the invasive species *W. chinensis*. Generally, invasive plants have faster growth rates and the ability to compensate for and maintain similar root/shoot ratios after damage (Ashton & Lerdau, 2008; Gard, Bretagnolle, Dessaint, & Laitung, 2013). In our study, some of the native species were more tolerant than the invasive species, likely due to the properties of the plants or other abilities of invasive species, such as herbivore resistance or allelopathy (Barton, 2016).

This study indicates that an allometric model provides a better approach than other methods for examining the relationship between biomass allocation and herbivory tolerance, investment in leaves is an important mechanism of tolerance, and investments in stems and roots do not improve tolerance in vines or creeping herbs. Additionally, the results indicate that investment in photosynthesis is related to the mechanisms used by plants to tolerate herbivory. Moreover, plants with invariant biomass allocation patterns may be more tolerant. Our experiment also revealed that the allometric exponent accurately reflects the effects of herbivory on biomass allocation patterns and can thus be used to assess the relationship between biomass partitioning pattern and herbivory tolerance. Therefore, the allometric model is more suitable than other methods for studying the mechanism of herbivory tolerance and is helpful for understanding the mechanics of herbivory tolerance. We studied the relationship between herbivory tolerance and biomass allocation with a different approach and different study species. Therefore, our results are different from those of other studies, and it is necessary to compare our results and methods with those of previous studies in the future. Because the allometric model removed the effect of plant size on the allocation pattern, the scope of this study included more than the responses of plants to herbivory, and it is important to determine the responses of plants to other circumstances and factors.

## CONFLICT OF INTEREST

None delared.

## AUTHOR CONTRIBUTIONS

ZXF, BMC, HXL, and SLP conceived and designed the experiments. ZXF and GHZ performed the experiments. ZXF and HXL analyzed the data. ZXF, BMC, and HXL wrote the manuscript.

## Data Availability

The data used to support the findings of this study are included in the supplementary information file.
